# Textile‐Based Ti3C2 MXene Capacitor by Laser Ablation Patterning

**DOI:** 10.1002/open.202500253

**Published:** 2025-08-05

**Authors:** Eugenio Gibertini, Ali Gokhan Demir, Riccardo Cesaro, Prisca Viviani, Luca Magagnin

**Affiliations:** ^1^ Department of Chemistry, Materials and Chemical Engineering “Giulio Natta” Politecnico di Milano Via Mancinelli 7 20131 Milano Italy; ^2^ Department of Mechanical Engineering Politecnico di Milano Via Giuseppe La Masa 1 20156 Milano Italy

**Keywords:** energy storage, fabric, flexible, gel polymer electrolyte, wearable

## Abstract

E‐textile technologies are quickly advancing, but the power supply is still one of the limiting factors, particularly for those integrated into textiles. There is a pressing demand for flexible textile‐based microdevices capable of storing and supplying energy. In this work, it is demonstrated that laser ablation (LA) can be conveniently used to achieve patterned thin film electrodes with interdigitated configuration on TPU‐coated cotton fabric to produce textile‐based energy storage units. Namely, Ti3C2 MXene (MX) electrodes were patterned via LA and coated with a LiCl based UV‐curable gel polymer electrolyte to produce a textile‐based flexible symmetrical capacitor. It is also shown that the LA process should be carefully designed to prevent electrode degradation during the process itself. The capacitance of textile‐based MX symmetrical capacitors (MX Sy‐Cs) ranged from 11.7 mF cm^‐2^ to 0.53 mF cm^‐2^ depending on the scan rate. By galvanostatic cycling at 100 µA cm^‐2^, the average capacitance was 2.03 mF cm^‐2^ with the C/C_0_ = 0.8 condition found after 9025 cycles. Moreover, an array of textile‐based MX Sy‐Cs is demonstrated to be compatible with low power textile energy storage applications.

## Graphical abstract

It is shown here that laser ablation can be effectively employed to pattern Ti3C2 MXene electrodes on TPU‐coated cotton fabric, creating high‐performance textile‐based capacitors. This technique allows for sub‐millimeter resolution, ensuring a compact and flexible design suitable for wearable applications. It is also shown that careful optimization of the laser ablation process is essential to prevent electrode degradation and maintain high electrochemical performance.

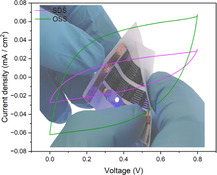

## 1 Introduction

Wearable electronics and internet of things (IoT) devices are increasingly becoming a part of our everyday life and are expected to play soon an indispensable role in many applications as sport‐tracking activities and healthcare.^[^ ^1^ ^,^ ^2^ ^]^ Among these, textile‐based electronics stand out as they can be seamlessly integrated into fabrics, making them comfortable for close contact with the human body.^[^ ^3^ ^]^ A number of advanced examples of textile‐based sensing, communication, luminescent, and energy harvesting units is present in the state‐of‐the art.^[^ ^3^, ^4^, ^5^, ^6^ ^–^ ^7^ ^]^ However, the development of energy storage devices capable of storing and supplying energy on demand to power other wearables is still challenging.^[^ ^8^, ^9^ ^–^ ^10^ ^]^ While both microsized and flexible batteries, capacitors, and hybrid devices are being explored, achieving sub‐millimeter resolution in electrode patterning remains a key challenge for fabricating small, wearable textile‐based energy storage units.^[^ ^11^ ^]^ In this context, thin‐film interdigitated electrode (IDE) configuration is a common and convenient choice in the design of planar and intrinsically flexible devices on nonrigid substrates as textiles.^[^ ^12^ ^,^ ^13^ ^]^ Limitations here arise in terms of manufacturing techniques since electrode patterning with high resolution (sub‐mm scale) is always a nontrivial step to fabricate small wearable devices. Most of the current advanced techniques employ additive‐manufacturing technologies that could be conveniently used to achieve patterned electrodes of different materials on a variety of substrates.^[^ ^14^ ^]^ For instance, masked sputtering is typically used to metal current collector deposition followed by subsequent coating step by electrochemical plating.^[^ ^15^ ^]^ Printing techniques as extrusion printing, screen‐printing, and inkjet‐printing allow good resolution patterning, even lower than 0.1 mm, but the formulation of inks is not trivial, usually posing some restriction in viscosity or particles size.^[^ ^14^ ^]^ In the last years, laser technologies have been standing out as, quick and versatile technique for high‐resolution patterning up to the submicrometer scale.^[^ ^16^, ^17^, ^18^ ^–^ ^19^ ^]^ According to the laser source and process parameters, laser irradiation can induce carbonization and/or oxidation of precursor material or selective ablation of some portion of the material coating. The former case is popularly employed in laser‐induced graphene (LIG) process.^[^ ^20^ ^]^ Electrodes for both flexible batteries and supercapacitors were produced by “laser‐writing” and graphenization of graphene oxide, polyimide (PI), nanocellulose, and other precursor materials that can be carbonized and converted to carbonaceous or graphenic‐like structures.^[^ ^21^, ^22^ ^–^ ^23^ ^]^ In the latter case, laser ablation (LA) can be used to selectively remove virtually any thin film coating to create the interelectrode spacing. As the two electrodes will have the same composition, this technique is very suitable for the manufacturing of symmetrical electrode energy storage devices as symmetrical capacitors (Sy‐Cs).^[^ ^18^ ^,^ ^24^ ^,^ ^25^ ^]^ Despite carbon‐based materials are the most commonly employed to fabricate Sy‐C, the class of 2D MXene (MX) materials is nowadays standing at the frontier of innovative yet popular material for energy storage purpose, including wearable devices.^[^ ^16^ ^,^ ^26^ ^,^ ^27^ ^]^ In particular, flexible Ti3C2Tx MX Sy‐Cs with IDEs were previously demonstrated and obtained through a variety of techniques, including selective LA pattering. For instance, Ti3C2 MX ink was sprayed on paper substrate and patterned by UV‐laser up to ≈0.2 mm resolution.^[^ ^28^ ^]^ After coating with acidic gel‐polymer electrolyte (H_2_SO_4_/PVA), the as‐obtained Sy‐C showed high areal capacitance of 23.4 mF cm^‐2^. In other cases, a combination of laser writing and LA processes was used to achieve planar Sy‐C of pure Ti3C2 MX or carbon/Ti3C2 composite electrodes. In fact, Cheng et al. demonstrated the advantage of irradiating a Ti3C2 coating with a 1030 nm femtosecond laser beam followed by the LA step to obtain the patterned electrodes.^[^ ^29^ ^]^ The first laser writing irradiation step was adopted to expand the interlayer spacing of the restacked Ti3C2 sheets through rapidly vaporizing interlayer water within MX film and increasing the ions accessibility and storage capability. The as‐obtained planar Sy‐C on PET flexible substrate showed a high areal capacitance of 145.2 mF cm^‐2^ at a scan rate of 20 mVs^‐1^, improving of 28% compared to the same device without expanded layers. On the other hand, in similar works Fu etal. demonstrated the feasibility of Sy‐C for wearable applications by the LIG of a GO/Ti3C2 composite coatings and subsequent ablation patterning using PI tape as substrate.^[^ ^30^ ^,^ ^31^ ^]^ In both cases, the areal capacitance ranged between 0.36 and 3.69 mF cm^‐2^. In overall, these studies confirmed the suitability of LA pattering for the easy and quick manufacturing of thin‐film planar Sy‐Cs. However, conventional polymeric substrates such as polyethylene (PET) and PI film were still employed that, despite their flexibility, are hardly compatible with fabrics and whose seamless integration in textiles is not trivial.

In this regard, we herein demonstrate that LA can be conveniently used for the manufacturing of true textile‐based capacitors for wearable energy storage. Taking advantage of the poor optical absorbance of the TPU layer to the employed laser wavelength, contrarily to the MX coating, we used a near infrared nanosecond‐pulsed fiber laser to selectively remove Ti3C2 MX layers from TPU‐coated cotton fabric to create patterned symmetrical electrodes. The MX electrodes with interdigitated design were subsequently coated with a UV‐cured gel electrolyte composed of 3 M LiCl dissolved in ethylene glycol and acrylamide as a cross‐linkable polymer matrix. The resulting textile‐based MX Sy‐Cs demonstrated optimal areal capacitance values, up to 11.7 mF cm^‐2^ and practical feasibility for energy storage and micropower applications.

## 2 Results and Discussion

### 2.1 LA Process Characterization

**Figure 1:**
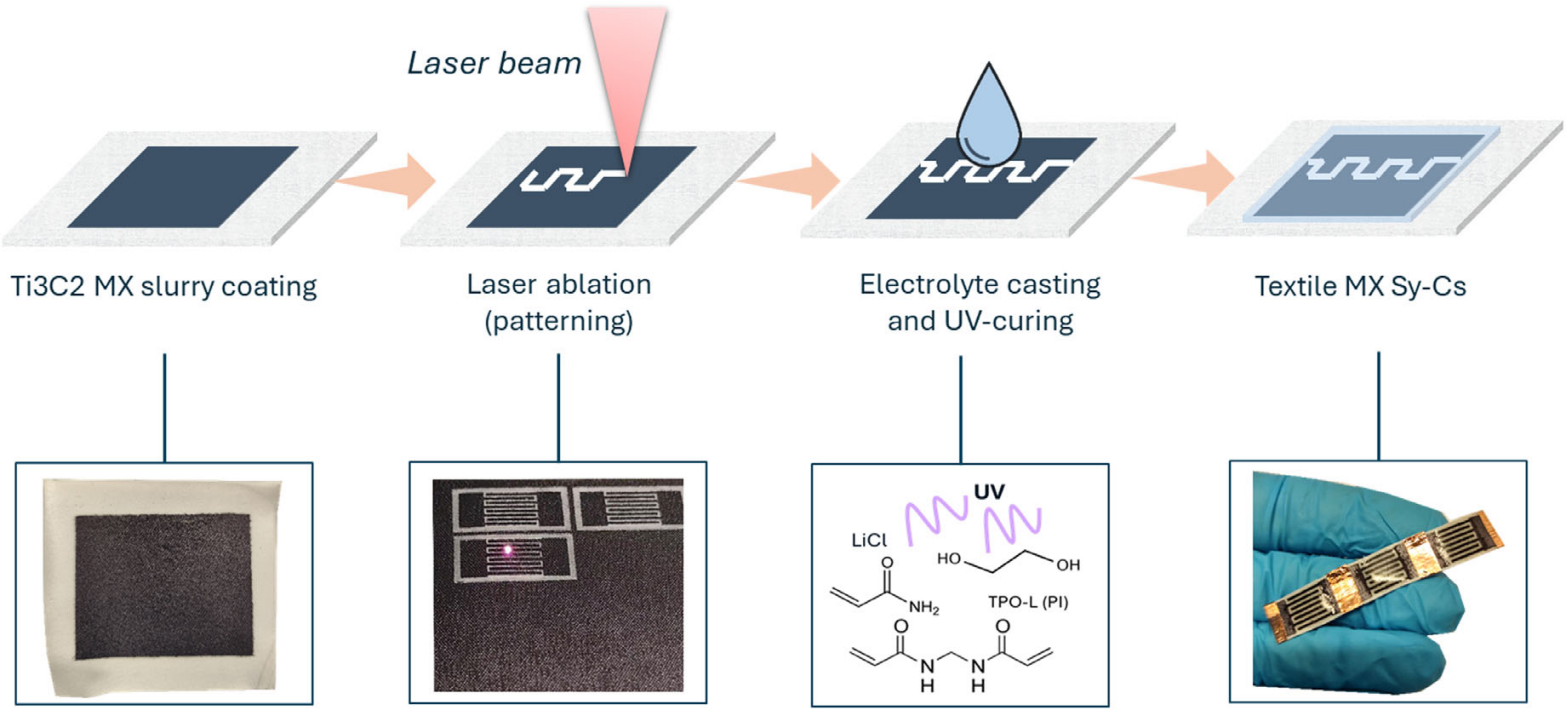
Schematic view of textile‐based Ti3C2 MX Sy‐Cs (MX sy‐C) preparation, with digital picture relative to each preparation step.

Ti3C2 MAX phase particles (Figure S1a, Supporting Information) were successfully etched in the HCl/HF mixture, producing the typical “open book” morphology as result of Al layers removal as shown by scanning electron microscope (SEM) image (Figure S1b, Supporting Information).^[^ ^32^ ^]^ According to the EDX analyses, the surface termination in multilayer MX were mostly oxygen and fluorine, with chlorine at low extent (Figure S1c, Supporting Information). After delamination step, individual 2D MX sheets were produced, as demonstrated by the [002] peak shift in X‐ray diffraction (XRD) patterns from 9.5° to 6.1° corresponding to a d‐spacing interlayer increase from 9.3 to 14.4 nm as result of intercalated H_2_O molecules and residual Li^+^ ions (Figure S2, Supporting Information). Moreover, the high order peaks almost disappeared for the free‐standing film obtained by vacuum filtration of delaminated Ti3C2 MX, meaning efficient restacking of the particles because of their 2D nature. In **Figure** **1**, the schematic representation of Ti3C2 MX textile capacitor preparation is presented. Cotton fabric was coated with a thin TPU layer as an airtight and waterproof shielding layer, capable of reducing the intrinsic fabric porosity, as is common practice in the textile industry. The MX mass loading after blade coating and drying was 1 mg cm^‐2^. After electrode patterning by LA, the final device was obtained by casting a liquid gel‐polymer electrolyte (GPE) precursor solution and crosslinking it in situ via UV irradiation, resulting in a shiny, transparent coating of GPE.

**Figure 2:**
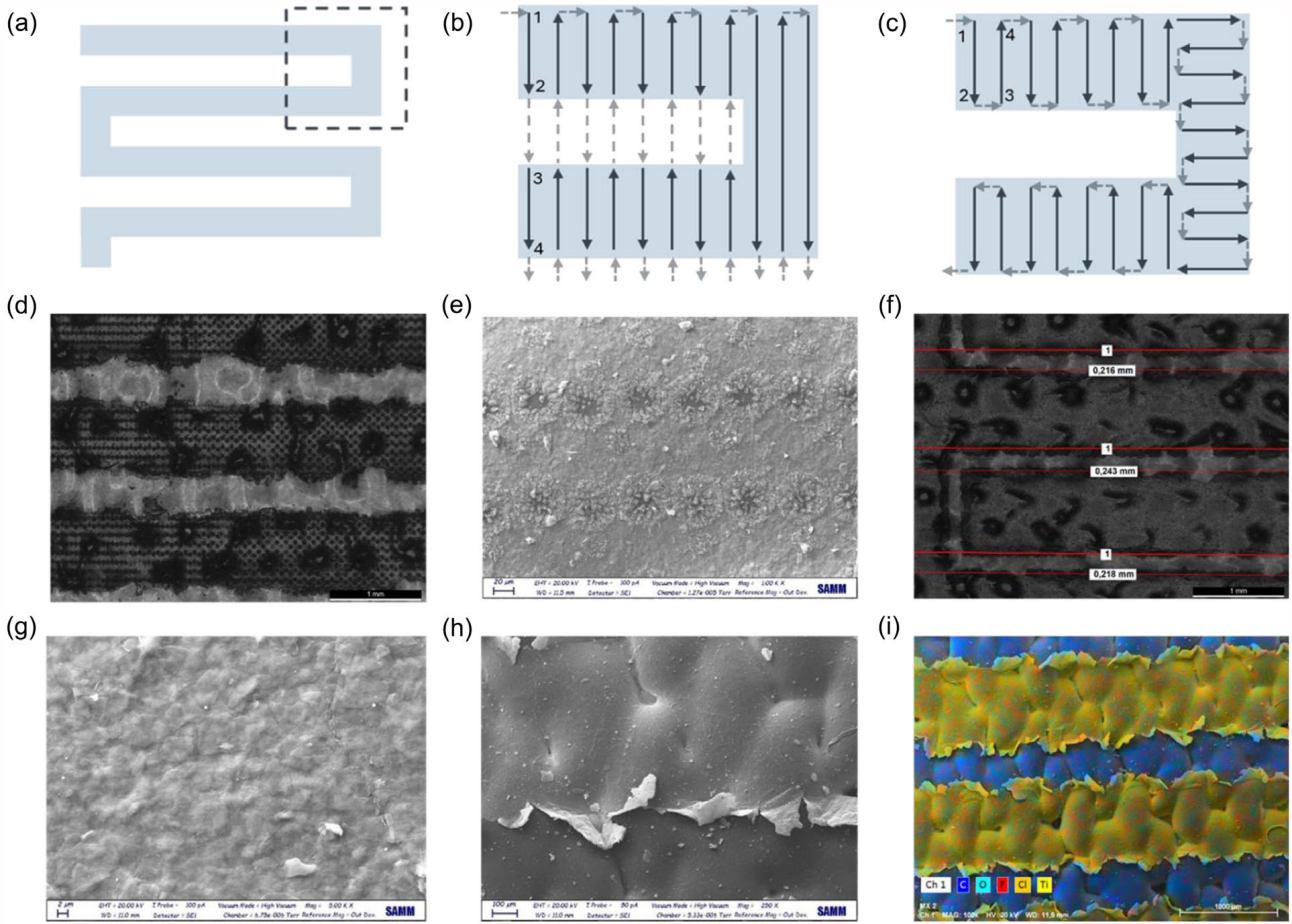
a) Schematic representation of the laser ablated pattern to create theIDE architecture; b) schematic representation from the dashed area in (a) of the laser movements according to the SDH; and c) OSS ablation mode. The blue arrows represent the laser emission movements, while the grey dashed arrows indicate the laser‐off movements. Stereoscopic and SEM images of d,e) the SDH and f,g) OSS laser patterned electrode. h) SEM and i) EDX mapping at the interface of ablation for the OSS patterned electrode are reported.

The pulsed laser source requires evaluating scan strategies for process development. A low‐energy seed laser is sent to an amplifier, increasing pulse energy to the desired level. The seed laser remains on, while amplification is controlled to modulate power. The amplification stage takes microseconds to rise and fall, releasing low‐energy pulses (<0.5 µJ) between stages. Though most metals and ceramics remain unaffected, highly absorbent materials may be partially machined. Two distinct scan strategies were adopted for the process as shown in **Figure** **2**a–c. The single direction hatching (SDH, Video S1, Supporting Information) strategy involved the scanning of the entire pattern following a single direction of hatching and scanning each vector row by row. The laser jumped between the scan regions as the amplifier was turned off. Such a strategy is often used as a generic solution to calculate the scan trajectory with ease. In the optimized sorting scan (OSS, Video S2, Supporting Information), the laser beam followed the pattern geometry without jumping between scan regions. Such solution adapts the scan trajectory to the pattern geometry, which can be implemented mathematically or manually by the programmer. The two strategies were investigated to assess the sensitivity of the material to laser irradiation with low energy to tackle the issues that may arise thereof. Stereoscopic and SEM images (Figure 2d,e) clearly show that the SDH mode damaged the Ti3C2 MX electrodes, producing a regular pattern of vaporization craters of ≈48 µm diameter in the nearby ablated area. Despite the low energy content of the pulses in the nonamplified region, the high optical absorptivity of Ti3C2 MX resulted in partially damaged regions along the jump trajectory. These small craters depict partial evaporation in the center and material remelting in the radial direction, confirming the sensitivity of the material to laser irradiation. However, within the ablation pattern, the surface was clean, free of residual MX matter, and did not show any substrate degradation, owing to the low optical absorption of the TPU‐coated white cotton substrate, as well as the potential local melting and subsequent solidification of the thin thermoplastic PU layer. On the other hand, such craters were not observed for samples produced by the OSS mode (Figure 2f,g), as the laser beam remained confined within the ablation pattern providing a more controlled ablation process. Setting the interelectrode distance to 0.1 mm by design, the actual ablation width resulted to be 0.225 mm. The relatively poor line fidelity was attributed not only to the pulse duration and beam size of the laser source but also to the few‐µm lateral size scale of the Ti3C2 MX bidimensional sheet, as shown by the SEM images in Figure S3a, Supporting Information. In fact, as shown in Figure 2h, at the interface of laser ablated area MX sheets randomly wrinkled and bent as the MX vaporization and shock wave partially expelled some excess MX material in the surroundings. As a result, the electrode strips surface at the ablation interface was evidently jagged, as shown by the EDX map in Figure 2, and the actual ablated portion was larger than the designed one. Moreover, the coating thickness was measured to be about 2.6 µm (Figure S3b, Supporting Information). Higher geometrical fidelity may possibly be achieved using ultrashort pulsed lasers and smaller beam sizes, which may come at an expense of reduced productivity.

### 2.2 Electrochemical Performance of Ti3C2 MX Symmetrical Capacitor

**Figure 3:**
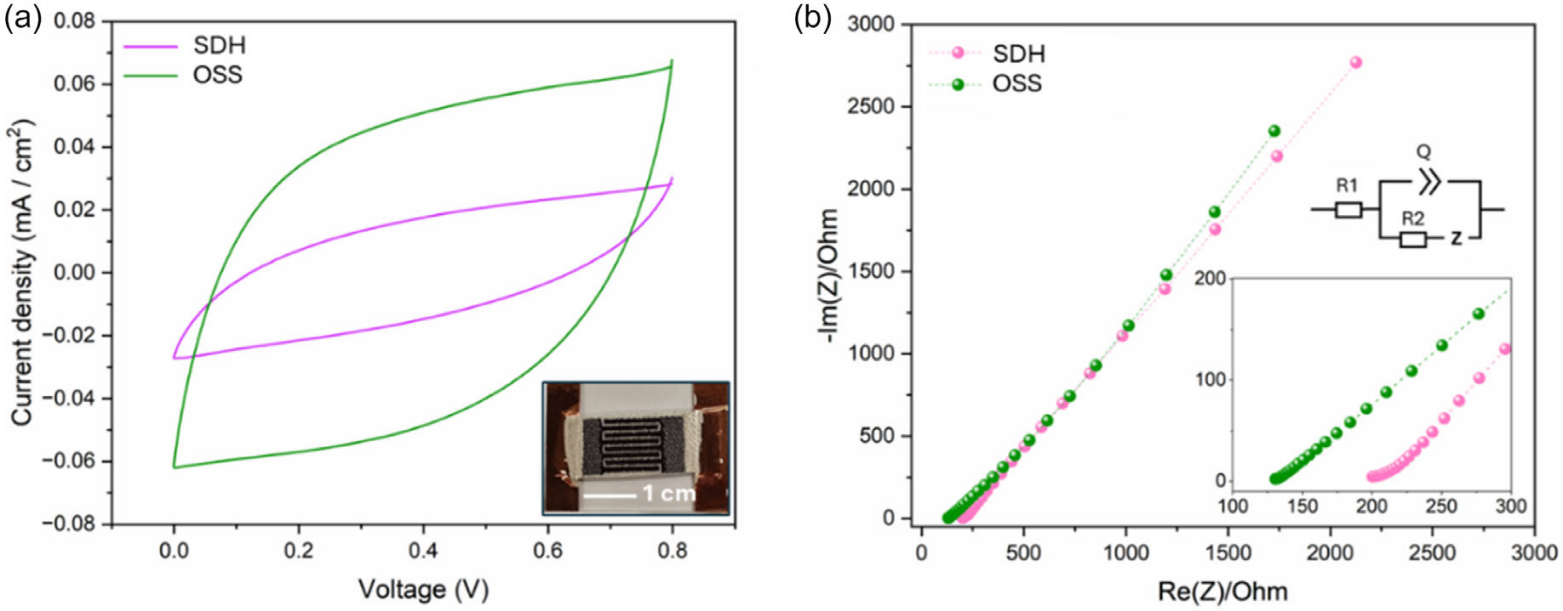
a) CV plots at 5 mVs^‐1^ for the textile‐based Sy‐Cs prepared by OSS and SDH LA mode, with digital picture of the typical Sy‐Cs tested samples (active area 1 cm^2^). b) Nyquist plot in the 100 kHz‐100 mHz range, with the equivalent circuit used to extrapolate the ESR (R1 in the equivalent circuit) from the linear sloped branch that can be fitted with a Randles circuit.

As shown in **Figure** **3**a, LA mode greatly affected the electrochemical performance of the as‐produced textile MX Sy‐Cs. Cyclic voltammetry (CV) shrank for the Sy‐C produced by SDH respect to the one by OSS, in the voltage window 0–0.8 V, corresponding to an areal capacitance of 2.44 mF cm^‐2^ and 7.76 mF cm^‐2^, respectively. The partial electrode vaporization and thermal degradation for patterned electrodes prepared by SDH mode induced higher electrodes impedance with detrimental effect on the capacitance. In fact, the calculated equivalent series resistance (ESR) was extracted from the Nyquist plots in Figure 3b, and it was calculated to be 209.2 Ω and 131.6 Ω for SDH and OSS mode, respectively. Moreover, the single sloped Nyquist plots at frequencies lower than 100 kHz were well representative of double‐layer capacitive behavior of the electrodes.^[^ ^33^ ^]^ These results suggest that other than laser source, ablation mode should be carefully designed to produce high quality patterned electrodes without affecting material properties. In overall, OSS ablation mode was selected and adopted for all the preparation of other samples.

**Figure 4:**
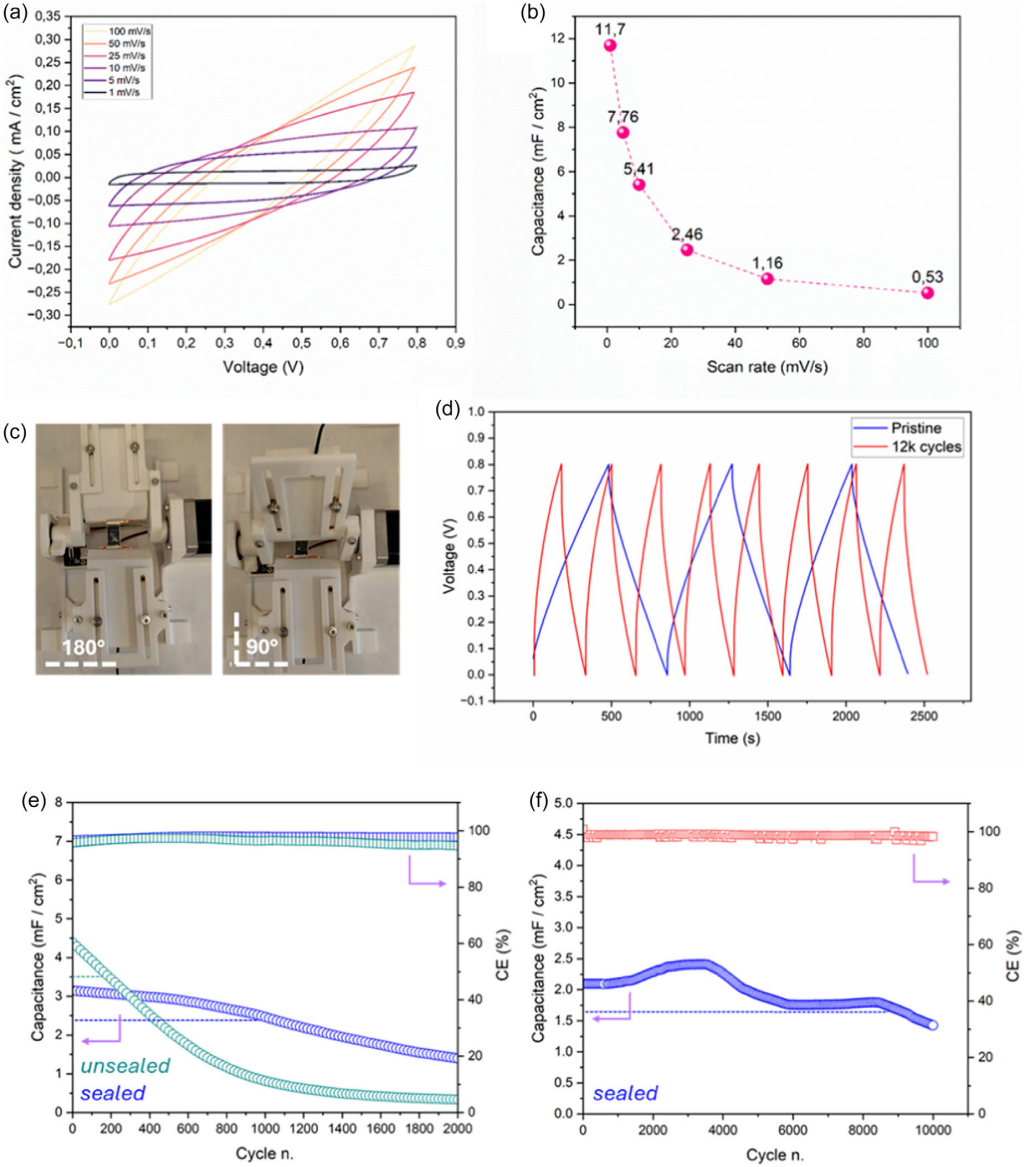
a) CV plots at increasing scan rate and b) corresponding capacitance as function of scan rate. c) Digital images of the bending tool and d) V‐t charge–discharge profiles before and after 12,000 bending cycles. Areal capacitance and CE of Sy‐Cs unsealed and sealed with the final coating of UV‐curable silicone elastomer, cycled at 50 and 100 µA cm^‐2^ e,f). The areal capacitance corresponding to the C/C_0_ = 0.8 condition is pointed out by the dashed lines.

Rate capability of the textile based Sy‐C was evaluated by CVs in the scan rate range 1100 mV s^‐2^ (**Figure** **4**a). The almost rectangular CV plot at low scan rate (1 mV s^‐2^), typical of double‐layer charge storage of Li+ in neutral solution for Ti3C2 electrodes is deformed and distorted as result of the increased polarization. Noteworthy, the as‐prepared Sy‐C were current collector‐free and the thin Ti3C2 MX layer acted both as current collector and charge storage material, simplifying the device preparation process but negatively affecting the ESR and, as consequence, power rate capability. The extrapolated areal capacitance quickly dropped from 11.7 mF cm^‐2^ at low scan rate (1 mV s^‐2^) to 2.46 mF cm^‐2^ at medium scan rate (25 mV s^‐2^) and up to 0.53 mF cm^‐2^ at 100 mVs^‐1^ (Figure 4b). These values are comparable to, but higher than, similar Sy‐Cs prepared with composite electrodes of GO/MX and PI/MX composites and acidic aqueous PVA‐basedGPE.^[^ ^30^ ^,^ ^31^ ^]^ When energy storage devices are designed for wearable application, in particular textile‐based one, the capability to withstand mechanical stimuli should be considered and evaluated, despite the current lack of standardized testing protocols that often push researcher in neglecting this kind characterization. Here, we evaluated the capacitance retention coupled with 90° bending cycles using a custom‐made bending tool (Figure 4c). In Figure 4d, the V‐t charge–discharge profiles in pristine condition and after 12,000 bending cycles are reported. As clear, bending deformation resulted in capacitance degradation more than halved from 5.96 to 2.51 mF cm^‐2^. In our previous work on inkjet‐printed textile MX capacitors, we observed similar results as consequence of bending deformations and we attributed this to the GPE/MX interface degradation or partial but cumulative overoxidation of Ti^3+^ to Ti^4+^ in MX of the positive electrode that is well known to occur at relatively low potential, slightly higher than 0 versus SCE.^[^ ^34^, ^35^ ^–^ ^36^ ^]^

Textile‐based Sy‐C obtained by LA patterning was galvanostatically cycled at 50 and 100 µA cm^‐2^ in the 0–0.8 V range without any mechanical deformation. In particular, the effect of a sealing layer was considered. At 50 µA cm^‐2^, the capacitance of the unsealed MX‐capacitor continuously decreased by cycling and quickly reached 80% of its initial value (C/C_0_ = 0.8) after just 201 cycles. On the other hand, the textile MX Sy‐C embodying a final sealing layer greatly improved the capacitance retention at C/C_0_ = 0.8 up to 990 cycles. Within the C/C_0_ = 0.8 conditions, the average areal capacitance, discharged energy and power were 2.21 mF/cm^2^, 0.13 and 6.49 μW cm^‐2^ for the unsealed device and 2.91 mF cm^‐2^, 0.11 and 10.46 μW cm^‐2^ for the sealed one. On the other hand, the final sealing coating slightly improved the average coulombic efficiency (CE) from 96.83 to 97.74%. In overall, all these improvements can be addressed to the sealing effect that was able to mitigate ethylene glycol solvent evaporation from GPE and molecular oxygen, and moisture permeation to the GPE and MX electrodes. Increasing the current to 100 µA cm^‐2^, the capacitance retention up to C/C_0_ = 0.8 greatly improved to 9025 cycles and the average CE was 98.62% while the average capacitance was 2.03 mF cm^‐2^. These results suggest that the capacitance fading could be mostly attributed to the partial, irreversible overoxidation of the MX positive electrode. In fact, generally, the higher the cycling current, the greater the cell polarization that in this case shifted the positive MX electrode electrooxidation to more positive potentials, approaching or overcoming the upper voltage cut‐off (0.8 V). This behavior is the main limitation of symmetrical electrode capacitors, in particular when the charge storage is not balanced between the two electrodes. This is typical for Ti3C2 MX, which usually shows poor charge storage properties at positive potentials.

### 2.3 Ti3C2 MX Sy‐Cs Array

**Figure 5:**
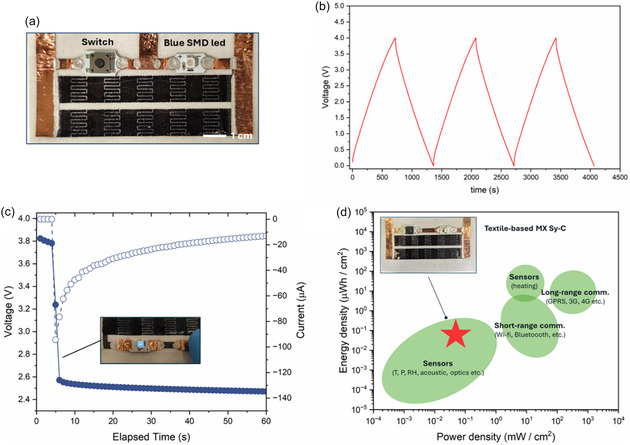
a) Digital image of the textile‐based array of MX Sy‐Cs integrated with a circuit comprising an SMD blue LED and a switch button. The total active area was 5 cm^2^. b) Charge–discharge V‐t profile of the array at 10 µA. c) Voltage and current profile during the blue LED powered by the MX Sy‐Cs array. d) Comparison of the areal power and energy density of the as‐produced textile‐based MX Sy‐C energy storage and power supply unit with respect to the energy and power density requirements of most typical technologies for wearables and IoT (readapted from^[^ ^38^ ^]^).

One of the main advantages of LA patterning is its easy adaptability for the automated fabrication of small devices, with no restrictions in the design other than the ablation resolution. We demonstrated here the possibility to use LA patterning for the one‐pot fabrication of textile‐based Sy‐c arrays for real energy storage and power supply applications in textile wearable electronics. Two parallelly connected arrays of five MX Sy‐Cs connected in series were designed and fabricated on the TPU‐coated cotton fabric (**Figure** **5**a). Both the transparent TPU layer as well as the white underlying cotton substrate poorly absorbed the 1064nm radiation and were neither affected nor degraded during the LA patterning of the MX capacitor arrays. The circuit was then integrated with a switch button and SMD blue LED. By galvanostatic cycling at low current density (10 µA cm^‐2^), the overall array capacitance was 2.12 mF (Figure 5b). Assuming the individual capacitor of the array equivalent to each other so that the equivalent capacitance for the five series connected devices is C_eq_ _.series _= n/C (n = 5) and the total array is C_eq.tot_ = 2C_eq.series_, the individual laser‐patterned MX Sy‐C had an areal capacitance of 10.6 mF cm^‐2^. In most IoT and wearable electronics applications, devices are meant to work in intermittent mode, so wearable power supplies should be able to deliver sufficient pulsed power.^[^ ^37^ ^,^ ^38^ ^]^ As a consequence, the instantaneous power delivered can be considered as well as the average power. We charged the textile‐based array of Sy‐C of Ti3C2 MX up to 4 V at 10 µA charging current, corresponding to 0.8 V of cut‐off potential on each individual capacitor. After a few seconds of rest, the blue LED was powered on by closing the circuit with the switch button. The array suddenly (after 1 s) delivered 303.6 µW of peak power with 94 µA current peak and voltage quickly dropped to the LED threshold voltage of 2.5 V (Figure 5c). Considering 1 min of power supply, the average current, power and total energy released by the array of textile‐based MX Sy‐Cs, were respectively 24.94 µA, 62.27 µW, and 1.03 µWh. According to typical power need of common IoT and wearable technologies, these values are compatible with low power demanding applications such as RFID tags, hearing aids, and optical, acoustic, and humidity sensors (Figure 5d).^[^ ^38^ ^]^ To fulfill the power need of short‐range communication technology, the proposed textile‐based Sy‐C array should be up‐scaled from 5 cm^2^ active area to at least 500 cm^2^, since the power density is mostly limited by the symmetrical device configuration. However, the as‐produced Sy‐C based power supply on TPU‐coated textile can be bent and is capable of withstanding various deformations (Video S3, Supporting Information). Despite our results in terms of electrochemical performance are inferior respect to other Ti3C2 MX Sy‐Cs obtained by various techniques,^[^ ^38^ ^]^ this works highlights thatLA can be conveniently used as patterning tool for the easy and quick preparation Sy‐Cs on TPU‐coated fabric for the development of true textile‐based capacitor devices.

## 3 Conclusion

The recent widespread adoption of lasers in different technologies is also impacting the microfabrication of wearable energy storage units and, in particular, electrochemical capacitor devices. The aim of this work was to demonstrate the feasibility of the LA pattering technique for the production of textile‐based Sy‐Cs. Ti3C2 MX was employed as electrode material and owing to the difference in optical absorbance between the transparent TPU and the dark MX layer, IDEs can be efficiently produced without affecting the underlying substrate. The resulting textile‐based MX Sy‐Cs show areal capacitance up to 11.7 mF/cm^2^, and long‐cycling tests highlight the importance of the sealing layer coating. Moreover, arrays of MX Sy‐Cs show practical feasibility for energy storage and power supply applications at low power density scale, with 303.6 µW of maximum power delivered when powering on a blue SMD LED. In overall, this work can inspire further advancements in textile‐based energy storage fabrication.

## 4 Experimental Section

4.1

4.1.1

##### Ti3C2 MXene Synthesis

Ti3C2Tx MX was synthesized through the well‐known HCl/HF mixed‐acid approach.^[^ ^39^ ^,^ ^40^ ^]^ Typically, 10 g of Ti3AlC2 MAX phase (Laizhou Kai Kai Ceramic Materials Co., Ltd,) was added to an etchant solution composed of 30 mL of HF, 120 mL of HCl, and 60 mL of H2O and stirred for 24 h at 35 °C. The obtained multilayered Ti3C2 was centrifugated and washed multiple times. For the delamination step, 2 g of the multilayered etched Ti3C2 was added to a solution of 40 mL of water and 2 g of LiCl and stirred for 24 h, followed by multiple centrifugations at 3500 rpm and finally delaminated placing the vial on a vortex mixer for 30 min at 2400 rpm. Polyacrylic acid (Sigma–Aldrich, average Mw ≈100,000) and sodium ascorbate (Alfa‐Aesar) were then added, respectively, as binder and antioxidation additive in a mass ratio of 10:1 and 30:1 respect to the solid Ti3C2 content in the viscous ink with a final MX concentration of ≈50 mgml^‐2^.

##### Laser Ablation Patterning

A white cotton fabric (≈0.15 mm thick) was coated with TPU thermoadhesive film (Platilon HU2, Covestro) using hot press. The viscous aqueous Ti3C2‐based ink was spread on the TPU‐coated cotton by doctor blade with a 100 µm blade gap. The coating was then dried at 50 °C for 2 h in a convection dryer. The LA patterning was carried out using a near‐IR fiber laser source (IPG YLP‐1/100/50/50, Cambridge, MA, USA) with the following key specifications: 54 W maximum average power, 250 ns pulse duration, 1064 nm wavelength, a frequency range of 20–80 kHz, and an M^2^ value of 1.7. The laser source was coupled to a galvanometric scanner head (TSH 8310 by Sunny Technology, Beijing, China), equipped with an F‐theta lens with a focal length of 100 mm (SL‐1064‐70‐100 from Wavelength Opto‐Electronic, Ronar‐Smith, Singapore), resulting in a focused beam diameter of 39 µm. The laser source pulsed using acousto‐optic Q‐switching. Through preliminary experiments, which are not reported here for brevity, the laser process parameters were chosen. The pulse energy was set to ablate the Ti3C2 MX coating throughout the complete thickness. The pulse overlap in space was managed via the control of the pulse repetition rate and the scan speed in order to allow for a complete removal over the scanned region. The hatch distance was set to ensure that no material was left unpatterned between the consecutive hatch lines. The adopted parameters for the ablation process that removed consistently MX throughout the material thickness was a pulse energy of 450 µJ and 20 kHz pulse repetition rate, resulting in 9 W average laser power. Laser energy above this value did not produce any improvement over the processing speed, while lower energy values resulted in incomplete removal and pattern inconsistencies. The laser was scanned at 1000 mms^−^^1^, producing a 50 µm distance between each consecutive ablated region on the material. A hatch distance of 0.05 mm was adopted, while the scanner was programmed to jump between consecutive scan lines at a speed of 2000 mms^−^^1^.

##### Symmetrical Capacitor Preparation

UV‐curable GPE was prepared according to our previously reported protocol with minor modification. Briefly, acrylamide and N,N ‐methylene‐bis‐acrylamide (mass ratio 20:1) were added along with TPO‐L photoinitiator (5% wt. respect to AM monomer) to a 3 M LiCl solution in ethylene glycol to have 10% wt. of cross‐linkable matrix. To prepare the Sy‐C, 100 µl cm^‐2^ of electrolyte were poured and homogeneously spread over the interdigitated area of the electrodes only. Finally, the GPE layer was produced by UV irradiation for 10 s using a UV lamp (395 nm, 50 W, TAOYUAN ELECTRON, Hong Kong). For sealed samples, a final protective coating was obtained by gently spreading on the GPE a small quantity of UV‐curable elastomer (SuperElastic, 3D materials) and curing it by UV irradiation for 10 s.

##### Characterization

MX particles and coating morphology were investigated through a SEM (Zeiss EVO 50 EP) equipped with an EDX detector (x‐sight detector, Oxford instruments, Abingdon, UK). The Microstructure of synthetized material was analyzed with an XRD microscope (model PW1830, K*α*1Cu = 1.54058 Å, Philips, Eindhoven, The Netherland). Images of the patterned electrodes were also acquired employing a stereoscope (Leica, Wild Heerbrugg).

Electrochemical characterization was performed on a Sy‐C featuring a 1 cm^2^ active area. CV and impedance spectroscopy (EIS) measurements were performed using a Biologic VSP‐300 potentiostat equipped with an EIS channel in the frequency range 100 kHz–100 mHz and 15 mV as pulse amplitude at open circuit potential. All measurements were performed at room temperature. Samples were galvanostatically cycled using a Neware BTS4000 cycler. The capacitance (F) of the printed capacitors was extracted from CV plots according to the equation C=∫IdV/(2ν∗ΔV) where *I* (mA) is the current, Δ*V* (V) is the voltage window, and *v* is the scan rate (mVs^‐1^). For galvanostatic charge–discharge experiments, the capacitance was calculated as C=I∫(dt/V(t)) . The discharged energy and power were calculated, respectively, according to the equations $E=0.5CV^{2}$ and P=E/t where *t* is the discharge time. To measure the drain current of theSy‐C array while powering on the blue LED, the array was parallelly connected in series to a multimeter sampling at 1 s (VC650BT SE, VOLTCRAFT).

## Conflict of Interest

The authors declare no conflict of interest.

## Supporting information

Supplementary Material

## Data Availability

The data that support the findings of this study are available from the corresponding author upon reasonable request.
