# Supplementary material for: Textile‐Based Ti3C2 MXene Capacitor by Laser Ablation Patterning
Source: ChemistryOpen. 2025 Aug 5;14(12):e202500253. doi: 10.1002/open.202500253 (PMC12680552; doi:10.1002/open.202500253)
Supplement: Supplementary file 1 — Supplementary Material [file OPEN-14-e202500253-s001.pdf]

Textile-Based Ti3C2 MXene Capacitor by Laser Ablation Patterning

E. Gibertini[1,*] A. G. Demir[2], R. Cesaro[2], P. Viviani[1], L. Magagnin[1]

[1]Department of Chemistry, Materials and Chemical Engineering "Giulio Natta", Politecnico di Milano

[2]Department of Mechanical Engineering, Politecnico di Milano, Milan, Italy

Corresponding: eugenio.gibertini@polimi.it

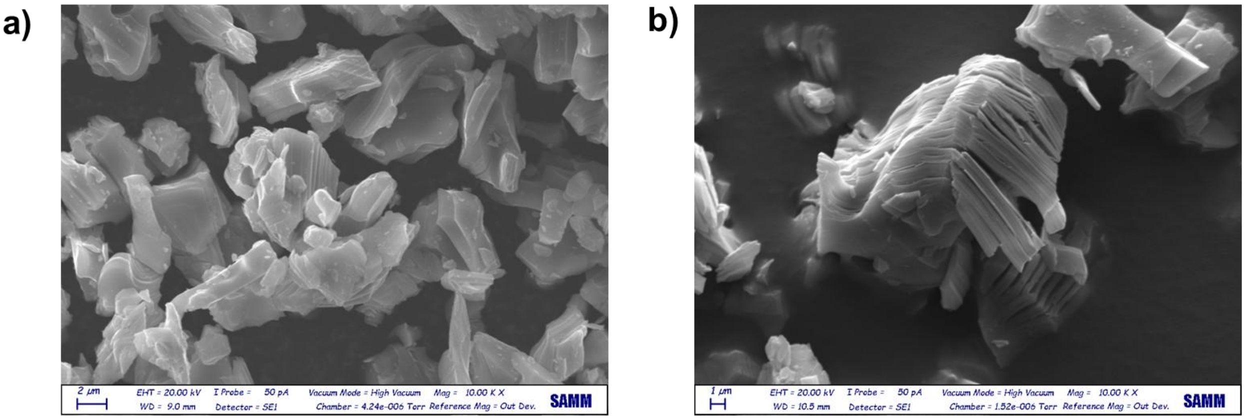

c)

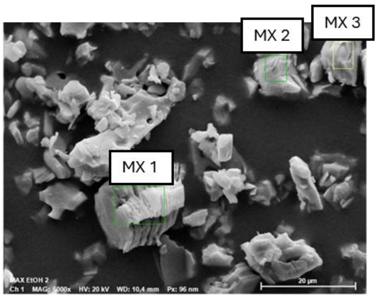

Atomic concentration [%]

Spectrum	Oxygen	Fluorine	Aluminium	Chlorine	Titanium
MX 1	24,65	19,41	0,53	2,24	53,16
MX 2	32,54	24,13	0,39	1,83	41,12
MX 3	31,15	25,17	0,32	1,93	41,43
Mean	**29,45**	**22,90**	**0,41**	**2,00**	**45,24**
Sigma	**4,21**	**3,07**	**0,11**	**0,21**	**6,87**
SigmaMean	**2,43**	**1,77**	**0,06**	**0,12**	**3,96**

Figure S1. SEM images of the pristine MAX TiAlC2 phase (a) and the etched multilayered MX particles (b). EDX quantitative analysis results with the corresponding area of analysis in the SEM image.

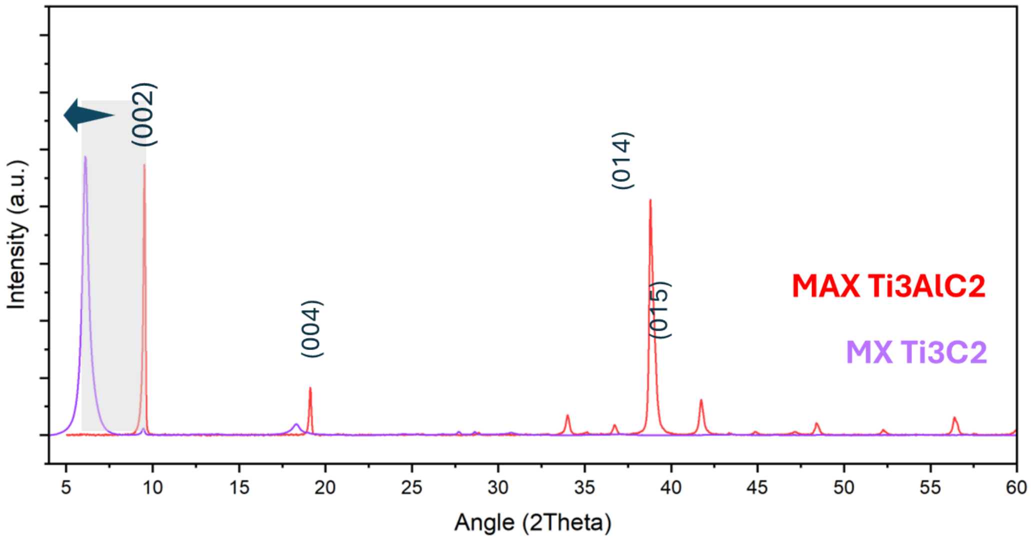

Figure S2. XRD pattern of pristine MAX phase powder and delaminated MX film obtained by vacuum filtration.

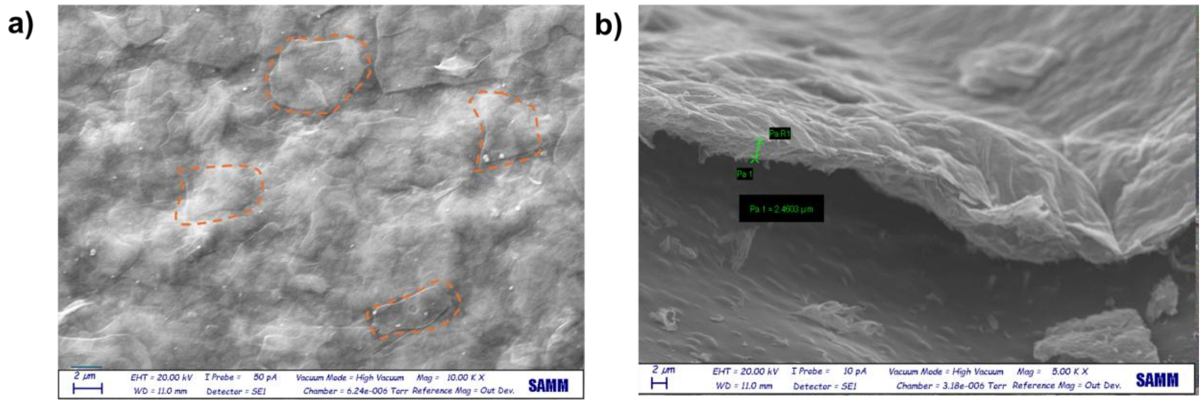

Figure S3. SEM image of the surface of the MX electrode patterned by OSS (a). The evident delaminated MX sheets are highlighted in the image. SEM image of the electrode cross-section at the interface of ablated area (b).
